# Supplementary material for: DPP: Deep predictor for price movement from candlestick charts
Source: PLoS One. 2021 Jun 21;16(6):e0252404. doi: 10.1371/journal.pone.0252404 (PMC8216512; doi:10.1371/journal.pone.0252404)
Supplement: Attachment — Submitted filename: Review of the Manuscript PONE-D-20-24642.pdf [file pone.0252404.s002.pdf]

Review of the manuscript PONE-D-20-24642_reviewer "***DPP: Deep Predictor for Price Movement from Candlestick Charts***" by Chih-Chieh Hung, Ying-Ju Chen

I will go directly to the points:

1- The manuscript uses the so-called well-known candlestick charts, which display price movements of a security, derivative, or currency for a specific period, and are popular charts for day traders. These charts are believed to be introduced by a Japanese rice trader *Munehisa Homma* in the 18th century.

Before going into the proposed idea by the manuscript, we should remind us that day traders, which can be human or not, are decision makers. Candlestick charts are there to help day traders in their decision making and thus, candlestick charts present information in aggregated, summarized form. Putting in another words, candlestick charts are like any similar (visual) aid that simplifies complex analysis behind the scene. Hence, nuances are lost in the candlestick charts.

The authors proposed that *instead* of reading the considerable body of numerical data from financial reports in the learning process, candlestick charts are used directly, as already seen in the abstract:

> we proposed a framework: deep predictor for price movement (DPP) by learning the candlestick charts instead of reading the considerable body of numerical data from financial reports.

Arguably, there is already here an inherently *danger* in this philosophy. Using generalization and simplifications as data sources introduce bias in the input data in the prediction purposes. In any prediction analysis, it is important to start granular and also analyze interactions among variables. By using visualization tools these aforementioned steps are already overlooked. It is in the analysis of the variables that we could aggregate some of them but not the other way around, i.e. not starting with already summarized outcomes and use them for prediction purposes.

Furthermore, we think for a moment what it is risk, we should remind us that it is in the tail of the distribution; although such events rarely happen, when they do, things will go terrible wrong. Unfortunately, those rare events tend to be undetected in those visual tools, unless tuned. Traders are supposed to take into consideration those risks, where you can "make it or break it".

Indirectly, the authors are claiming that candlestick charts have the capacity of resuming important information needed for the task at hand, which is price movement, and the rest of the information can be considered random. This is a hefty claim.

The authors are expected to explain the implications of the proposed model, clarifying the above statements in this review and link it to existing *theories* in the revised manuscript.

2- It seems to be a typo in "filed".

> **2.1 Deep Learning Approaches for Time Series Forecasting**
>
> Time series forecasting problems have been investigated in the filed of machine learning. Recurrent Neural Networks (RNN), a variant of neural network, is one of the

3- This is read in page 5/15:

> Secondly, the definition of price movement in this paper takes the mean and variance of prices of k-day history into account, rather than simply uses the difference of prices of the k-th day and the $(k+1)$-day, which can better represent the trend of the price movement.

3a) Please, provide evidence *in this paper* that the first two central (statistical) moments are better than "the difference of prices of two consecutive days".

3b) How come just the first two central statistical moments are chosen? Why not high-order moments?

4- A follow-up of the above point 3: Although any *daily* price movement method is useful for *daily* trading, the current methodology used statistical moments and not days in the calculations which opens the question: How this change in the aforementioned usage of statistical moments affects its implementation? Could this method be useful beyond the daily trading, and in so for how long?. I'm also asking this because in Table 2, results are presented by years, like the method is good for the long run as well, albeit I wonder if this has been sufficiently clarified. After all, short term strategy or daily trading have more chance to be more successful than the long-term prediction. Please, clarify all this. Thanks.

4- This is read in page 5/15:

> Thirdly, the predictor of the price movement is based on GRU, rather than 1D-CNN, which is more intuitive and brings higher accuracy in nested cross-validation.

In any prediction modeling, the standard praxis is to test several approaches. Please, chose more than one model, say 3 of them, and compare their results and their interpretations. Furthermore, implement previously classical known methods, *besides the already IEM model*, and compare all those methods and their interpretations. This is extremely important to determine any plausible improvement.

5- There is a typo in "n this section"

4.2 Performance Evaluation

n this section, we examine the performance of the proposed DPP based model and the

6- Table shows these results

Year	Experiment DPP	IEM
2007	79.76%	82.80%
2008	82.28%	84.94%
2009	78.47%	73.37%
2010	77.46%	78.75%
2011	76.34%	80.77%
2012	84.09%	82.06%
2013	86.00%	78.52%
2014	79.68%	77.49%
2015	82.23%	81.63%
2016	81.86%	79.42%
Average (2007-2016)	80.82%	79.98%
Average (2012-2016)	82.78%	79.83%

Table 2. Accuracy of DPP and IEM for each year

It can be argued that the differences are marginals, i.e. 3%, as claimed, ***when the 2012-2016 data are analyzed***. It can be argued that 3% can be within a margin of error, based on the chosen data and its time frame. The authors focus on the 3% allegedly improvement in that time frame as read here:

> However, DPP outperfoms IEM by about 3% when the 2012-2016 data are used.

but avoid such comparisons about other time frames using the same data. When does the method is not better? This could reinforce our suspicious that the "3% claim" are just random events. The original method is usually used for daily trading but results are presented in years. Thus: What about other data sources and presented those results and comparisons as well as in table 2?

Please, use the same data source but in different time frame. Also, use another data source within the same *and* different time frames. Compare those results in a table, similarly to table 2. At this stage, recall to implement several methods as pointed out in point 4 above. This should be part of the so-called back-testing, model comparison and sensitivity analysis as well.

Also, it is good to remind the authors that it is OK to reach a conclusion that the proposed method is not significantly better than the previous one, even if marginally better in some cases than previous methods. Those works are also publishable as well.

7- As pointed out by the authors, because "*deep-learning models have more parameters and require more data to tune these parameters to improve performance*" the authors have to be more explicitly in presenting those (hyper)parameters and their evolutions and how they have been selected. Graphical evolution of (hyper)parameters, their selection procedures, etc should be presented in the paper.

In case the authors wonder about such "evolution" of (hyper) parameters I'm refereeing to, two examples (of another method) are presented, just for guidance:

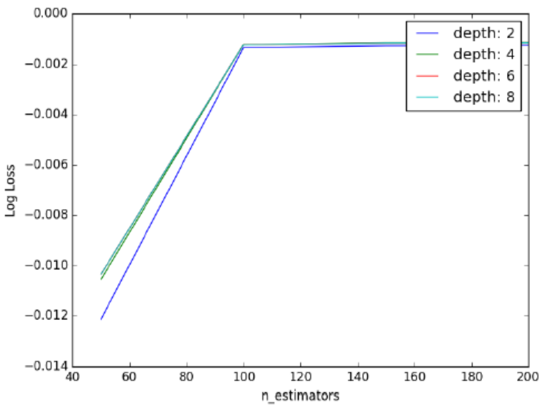

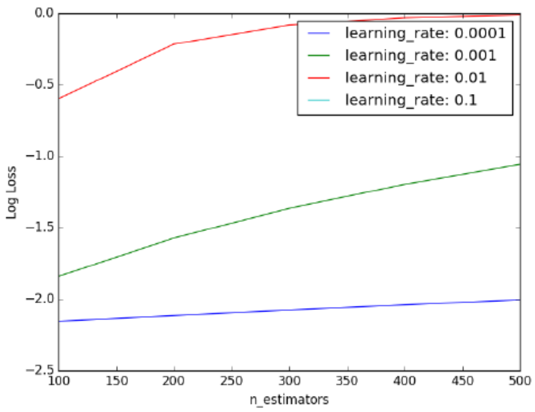

8- Please, include two figures, a ROC and a Precision-recall, containing all the tested methods, not only DPP and IEM but others, as requested above, as well as a table similar to table 3.

9- Section 4.3, is called sensitivity analysis. However, sensitivity analysis implies the study of a model exposed to different input variables to determine how target variables are affected based on changes in other variables known as input variables. Certainly, it can be argued that (hyper) parameters can be modified, changing the outcome but one of the main questions in the manuscript is to determine the model sensitivity to other input data as well, and compare this model sensitivity to other models' sensitivity. As points out above, other advanced models should be tested extensively as well together with different data sources and time frames.

10- This is read in the early pages:

In order to assess the performance of DPP, an extensive study of experiments is :onducted using the 15-year trading data of Taiwan Stock Exchange Capitalization Neighted Stock Index. The result shows that DPP outperforms the existing predictive

which can be argued it contains a complete economic cycle, at least from the Western economic crisis perspective back to “bull markets”. Taiwan had a deep as well around 2008 as seen in the picture below. Please, analysis, both Asian and Western stock markets and discuss the model outcome and performance, together with other models, as repeated requested above.

Some models are good for certain periods than others and it is a good idea to get a grip of the propose model “best time to be used” within an economic cycle, if it has any. In the discussion, please, include which model is good in which period within an economic cycle, etc. Still, the above 3% claim cannot necessarily be attributed that the market was particularly good to predict (as seen below in the figure); other reasons may be behind such results. Therefore, comparison with other models and data sources and time frame could clarify the issue.

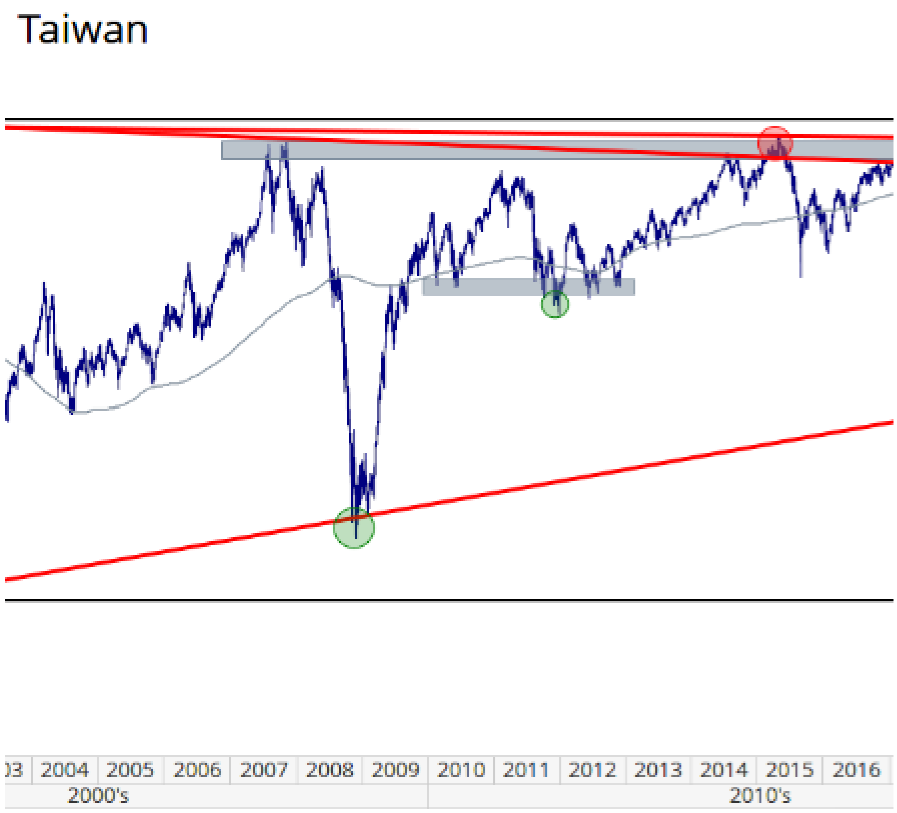

11 - Please, plot the outcomes of the methods to their targets, to visualized claims like “achieves higher accuracy”.

Thanks

Recommendation: **Major revision** of the submitted manuscript.

Thanks.
