# Supplementary material for: DPP: Deep predictor for price movement from candlestick charts
Source: PLoS One. 2021 Jun 21;16(6):e0252404. doi: 10.1371/journal.pone.0252404 (PMC8216512; doi:10.1371/journal.pone.0252404)
Supplement: Attachment — Submitted filename: response letter.pdf [file pone.0252404.s003.pdf]

Response to Reviewers' Comments: [PONE-D-20-24642] - [EMID:aed95d27d871ef1a]

We would like to thank the editor and the two reviewers for their constructive comments, which significantly improved our manuscript. In this document, we detail how we have addressed all the comments made by the editor and the reviewers. For the editor's/reviewers' convenience, we reproduce each comment in a grey box, then provide our response in bold. Furthermore, we highlight the changes in the manuscript in blue, with the exception of minor edits pertaining to typos and language issues.

Response to the Editor's Comments

> Thank you for submitting your manuscript to PLOS ONE. After careful consideration, I feel that it has merit but does not fully meet PLOS ONE's publication criteria as it currently stands. Therefore, I invite you to submit a revised version of the manuscript that addresses the points raised during the review process.
>
> I agree with the reviewer 2 that there are some weak points in this manuscript that seriously difficult the future publication. However, I have decided to give the authors the opportunity of revising the manuscript, but all concerns must be properly addressed.
>
> Why the authors consider that candlestick charts have the capacity of resuming all important information needed for prediction of price movement? Why the rest of the information can be considered random? How Candlestick charts have the ability of selecting the proper information?
>
> By the other hand, model performance in different scenarios should be also presented as well as comparison with others state of the art.
>
> I agree also with the reviewer that excess of performance could be considered as casual, so I consider that there are serious doubts that the conclusions are fully supported by the results of your experiment.

In this substantial revision, we have addressed all the reviewers' comments. We summarized the changes made in the following.

A. **We rewrote the introduction and modified the related work section to include recent related work and discuss how our approach is better. In addition, we included literature that supports candlestick charts having the capacity of resuming important information needed to predict the price movement. Literature regarding analysis using candlestick charts has explored the capabilities of using candlestick charts to predict the stock market prices. A candlestick chart contains low price, open price, high price, close price on a day which are the summary statistics of the daily price while other machine learning methods summarize data using different algorithms and they all somehow focus on important features in the data. In our case, the relative position of few neighboring candle sticks is the important feature which can represent several patterns for predicting the trend of price movement. In the field of machine learning, the predictors used could be subjective depending on the availability of data source while**

there exist some confounding variables that would affect the response variable and we usually leave the effect from those confounding variables as random.

B. **We included two additional baseline approaches: the additive approach Prophet, and the recurrent neural network based approach LSTM besides IEM and one additional data set Nikkei 225 from Tokyo Stock Exchange to reinforce the quality of the manuscript and compared the result from these baseline approaches with our proposed method.**
C. **ROC curves and Precision-recall curves for all models are included and the corresponding comparison is included in the subsection 4.2 Performance Evaluation.**

We thank you for your comments and feedback that helped us to significantly improve our manuscript.

Reviewer #1

1) Please review more recent related work and discuss how your approach is better.

Thank you for your careful review of our paper. We have included more recent related work in the introduction and related work sections in the manuscript. The introduction section was re-written based on the recent work in the literature. We also discussed how our approach is better by including one additional dataset and comparing the proposed method with 3 baseline approaches: IEM, Prophet, and LSTM and updated the results in the section 4: Experimental Results. The ROC curves and Precision-recall curves for different models were provided to support that our approach is better than other baseline approaches.

2) Explain in depth what deep learning architecture did you used.

Thank you for your suggestion. We have explained in depth about the deep learning architecture we used in Section 3: Deep Predictor for Price Movement in the manuscript. The architecture of CNN-Autoencoder is explained in Section 3.2 and is shown in Fig 4 with the corresponding parameters, such as the number of filters, the filter size, the stride size, the activation function, the pool size and so on. The architecture of GRU is explained in Section 3.3 and is shown in Fig 5 as well with corresponding parameters, such as the use activation function, number of hidden features.

3) Compare the end results with other related work.

Thank you for your suggestion. We have added the comparison of the end results with other related work in the manuscript. Table 1 and Table 2 in the manuscript report the average of accuracy values obtained from five randomly selected training and test data sets corresponding to different models based on the TX dataset and the NI225 dataset, respectively. Figure 7 and Figure 8 show the ROC and Precision-recall curves of DPP against the other baselines using the

TX dataset and the NI225 dataset, respectively. The comparison has been made in the subsections 4.2.1 and 4.2.2.

We thank the reviewer for the suggestions that helped us to significantly improve our paper.

Reviewer #2

Thank you for your careful review of our paper. We appreciate your comments and suggestions. Below, we provide our point-by-point response to your comments.

1- The manuscript uses the so-called well-known candlestick charts, which display price movements of a security, derivative, or currency for a specific period, and are popular charts for day traders. These charts are believed to be introduced by a Japanese rice trader *Munehisa Homma* in the 18th century.

Before going into the proposed idea by the manuscript, we should remind us that day traders, which can be human or not, are decision makers. Candlestick charts are there to help day traders in their decision making and thus, candlestick charts present information in aggregated, summarized form. Putting in another words, candlestick charts are like any similar (visual) aid that simplifies complex analysis behind the scene. Hence, nuances are lost in the candlestick charts.

The authors proposed that *instead* of reading the considerable body of numerical data from financial reports in the learning process, candlestick charts are used directly, as already seen in the abstract:

> we proposed a framework: deep predictor for price movement (DPP) by learning the candlestick charts instead of reading the considerable body of numerical data from financial reports.

Arguably, there is already here an inherently *danger* in this philosophy. Using generalization and simplifications as data sources introduce bias in the input data in the prediction purposes. In any prediction analysis, it is important to start granular and also analyze interactions among variables. By using visualization tools these aforementioned steps are already overlooked. It is in the analysis of the variables that we could aggregate some of them but not the other way around, i.e. not starting with already summarized outcomes and use them for prediction purposes.

Furthermore, we think for a moment what it is risk, we should remind us that it is in the tail of the distribution; although such events rarely happen, when they do, things will go terrible wrong. Unfortunately, those rare events tend to be undetected in those visual tools, unless tuned. Traders are supposed to take into consideration those risks, where you can "make it or break it".

Indirectly, the authors are claiming that candlestick charts have the capacity of resuming important information needed for the task at hand, which is price movement, and the rest of the information can be considered random. This is a hefty claim.

The authors are expected to explain the implications of the proposed model, clarifying the above statements in this review and link it to existing *theories* in the revised manuscript.

Thank you for your detailed comment and suggestions.

There has been lots of work in the literature that explored the capabilities of using candlestick charts to predict the stock market prices. We have added several recent publications to support the motivation of the study in both introduction and related work sections in the manuscript. The entire introduction section was rewritten and the subsection 2.2 Analysis of Candlestick Charts has been modified to clarify the rationale for using candlestick charts to predict stock prices.

2- It seems to be a typo in "filed".

2.1 Deep Learning Approaches for Time Series Forecasting

Time series forecasting problems have been investigated in the filed of machine learning. Recurrent Neural Networks (RNN), a variant of neural network, is one of the

Thank you for your careful review of our paper. We have fixed the typo.

3- This is read in page 5/15:

Secondly, the definition of price movement in this paper takes the mean and variance of prices of k-day history into account, rather than simply uses the difference of prices of the k-th day and the $(k+1)$-day, which can better represent the trend of the price movement.

3a) Please, provide evidence *in this paper* that the first two central (statistical) moments are better than "the difference of prices of two consecutive days".

3b) How come just the first two central statistical moments are chosen? Why not high-order moments?

Thank you for bringing highlighting these important questions.

(3a) In our study, we define the response variable by considering the average of previous k-day close prices (moving average) and the corresponding standard deviation. The idea of the definition was based on Bollinger Bands 1992 [1], a widely-used technical analysis tool to give a relative definition of high and low prices of a market, allowing traders to vary the response of the chart to the magnitude and frequency of price changes. If the close price on the d-th day is more than 1 standard deviation above the moving average, the close price is defined to be moving up, otherwise the close price is moving down. The movement direction decided using such a definition considers the overall distribution of the close prices in the past k days and it aims to decide if the price is going up compared to the close prices in the past k days, not just the previous day. If the distribution of the close prices is unimodal and symmetric, then about 68% of the time the d-th day close price will be within one standard deviation of the moving average. The definition indicates if a close price is higher than the close prices in the same distribution more than 84% of time (16%+68% based on the empirical rule of a normal model),

we consider this close price is a real increase, not an increase due to sampling errors. Since the price movement in a single day is usually highly associated with the prices in the previous few days, using the difference of prices of the k-th day and the (k+1)-day doesn't take into account the effect from the past multiple days. Latane and Rendleman 1976 [2] have shown that the standard deviations implied in actual call option prices contain useful information for predicting future stock price variability.

[1] Bollinger J. Using bollinger bands. Stocks & Commodities. 1992;10(2):47-51.
[2] Latane HA, Rendleman Jr RJ. Standard deviations of stock price ratios implied in option prices. The Journal of Finance. 1976;31(2):369–381.

(3b) In statistics, the standard deviation is a measure of dispersion which is used with the mean value to determine how unusual a value is in the data. For example, if the price $C_{d_1} = \mu + 0.5\sigma$**, this close price is only about 0.5 standard deviations above the mean price of the previous k days and we wouldn't consider this as a real increase while** $C_{d_2} = \mu + 1.5\sigma$ **indicates a real increase based on the definition as the price is about 1.5 standard deviation above the moving average. Therefore, other statistical moments are not necessary to be included in this situation as we just need to know how close the price is toward the mean price of the past k days.**

> 4- A follow-up of the above point 3: Although any daily price movement method is useful for daily trading, the current methodology used statistical moments and not days in the calculations which opens the question: How this change in the aforementioned usage of statistical moments affects its implementation? Could this method be useful beyond the daily trading, and in so for how long?. I'm also asking this because in Table 2, results are presented by years, like the method is good for the long run as well, albeit I wonder if this has been sufficiently clarified. After all, short term strategy or daily trading have more chance to be more successful than the long-term prediction. Please, clarify all this. Thanks.

Thank you for raising these important questions.
In Table 1 (original Table 2), the result was presented by years since the daily trading data for the entire year was used as the test data. That is, each accuracy value reported in Table 1 is the proportion of correct predictions made by a prediction model for the entire year. Therefore, the accuracy reported is based on the yearly performance of a predictor. Since short-term strategy may have more chance to be more successful, we believe that the performance for the entire year is proper to represent the performance of a predictor. There exists the potential to extend the study to a long run prediction as the response variable here is defined by the moving average and the corresponding standard deviation. However, it is beyond the scope of the study in this manuscript. We appreciate the reviewer's comments and suggestions about this and will look forward to exploring such a potential as a future direction.

> 5- This is read in page 5/15:

Thirdly, the predictor of the price movement is based on GRU, rather than 1D-CNN, which is more intuitive and brings higher accuracy in nested cross-validation.

In any prediction modeling, the standard praxis is to test several approaches. Please, choose more than one model, say 3 of them, and compare their results and their interpretations. Furthermore, implement previously classical known methods, besides the already IEM model, and compare all those methods and their interpretations. This is extremely important to determine any plausible improvement.

Thank you very much for the valuable suggestions! We have included additional two baseline approaches Prophet and LSTM besides IEM in comparison with our proposed DPP approach. The overview of these two approaches is included in Section 4.1: Settings and Datasets. The comparison of their results and the corresponding interpretations are updated in Section 4.2: Performance Evaluation. In addition, another dataset Nikkei 225 from Tokyo Stock Exchange (denoted as NI225) is added to explore the capability of the proposed DDP framework compared to other approaches.

6. There is a typo in "n this section"

4.2 Performance Evaluation

n this section, we examine the performance of the proposed DPP based model and the

Thank you for your careful review of our paper. We have fixed the typo.

7- Table shows these results

Year	Experiment	
	DPP	IEM
2007	79.76%	82.80%
2008	82.28%	84.94%
2009	78.47%	73.37%
2010	77.46%	78.75%
2011	76.34%	80.77%
2012	84.09%	82.06%
2013	86.00%	78.52%
2014	79.68%	77.49%
2015	82.23%	81.63%
2016	81.86%	79.42%
Average (2007-2016)	80.82%	79.98%
Average (2012-2016)	82.78%	79.83%

Table 2. Accuracy of DPP and IEM for each year

It can be argued that the differences are marginals, i.e. 3%, as claimed, when the 2012-2016 data are analyzed. It can be argued that 3% can be within a margin of error, based on the chosen data and its time frame. The authors focus on the 3% allegedly improvement in that time frame as read here:

However, DPP outperfoms IEM by about 3% when the 2012-2016 data are used.

but avoid such comparisons about other time frames using the same data. When does the method is not better? This could reinforce our suspicious that the “3% claim” are just random events. The original method is usually used for daily trading but results are presented in years. Thus: What about other data sources and presented those results and comparisons as well as in table 2?

Please, use the same data source but in a different time frame. Also, use another data source within the same and different time frames. Compare those results in a table, similarly to table 2. At this stage, recall to implement several methods as pointed out in point 4 above. This should be part of the so-called back- testing, model comparison and sensitivity analysis as well.

Thank you very much for raising the concerns and providing the suggestions here.

We have updated Table 1 and Table 2 by adding two additional approaches, Prophet and LSTM, using the TX dataset and the NI225 dataset. While the 3% could be within a margin of error based on the chosen data and its time frame, it has shown that the accuracy values of DPP are consistent higher than the accuracy values of IEM since 2012 when coinciding the TX dataset. It is evident that DPP outperforms three baseline models when the NI225 dataset is used based on the result reported in Table 2.

We have included the discussion of the performance of DPP and IEM using the TX dataset but in a different time frame in the third and the fourth paragraph in Section 4.2.1: Evaluation of TX dataset. A similar discussion using the NI225 dataset is also provided in the third paragraph in Section 4.2.2: Evaluation of the NI225 dataset. The model comparison as well as sensitivity analysis based on the NI225 dataset were completed and added in the manuscript in Section 4: Experimental Results.

8- As pointed out by the authors, because “deep-learning models have more parameters and require more data to tune these parameters to improve performance” the authors have to be more explicitly in presenting those (hyper)parameters and their evolutions and how they have been selected. Graphical evolution of (hyper)parameters, their selection procedures, etc should be presented in the paper.
In case the authors wonder about such “evolution” of (hyper) parameters I’m refereeing to, two examples (of another method) are presented, just for guidance:

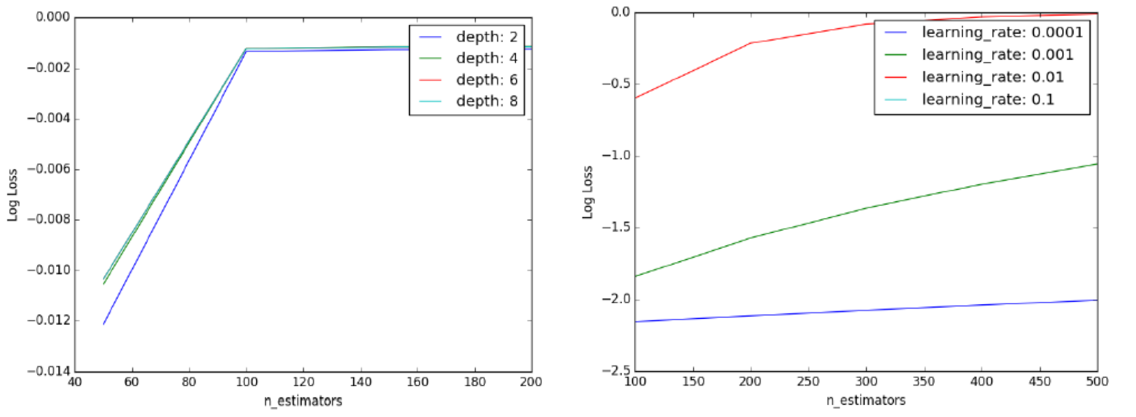

Thank you for the suggestions. We have added Figure 10 and Figure 11 to present the changes of accuracy with hyper-parameters varied using the TX and the NI225 datasets, respectively. The corresponding explanation and comparison are also updated from the third paragraph to the last paragraph in Section 4.3 in the manuscript.

9- Please, include two figures, a ROC and a Precision-recall, containing all the tested methods, not only DPP and IEM but others, as requested above, as well as a table similar to table 3.

Per your request, the ROC and Precision-recall curves based on DPP, IEM, Prophet, and LSTM are provided in the manuscript as shown in Figure 7 and Figure 8. We have removed Table 3 since ROC and PR curves are informative to show the prediction performance of different models.

10- Section 4.3, is called sensitivity analysis. However, sensitivity analysis implies the study of a model exposed to different input variables to determine how target variables are affected based on changes in other variables known as input variables. Certainly, it can be argued that (hyper) parameters can be modified, changing the outcome but one of the main questions in the manuscript is to determine the model sensitivity to other input data as well, and compare this model sensitivity to other models' sensitivity. As points out above, other advanced models should be tested extensively as well together with different data sources and time frames.

Thank you for the suggestion. In the second paragraph in Section 4.3, we have added the experiments and discussion regarding model sensitivity where we presented how the accuracy of DPP and IEM in the TX dataset and the NI225 dataset changes in two different markets in the same time period.

11- This is read in the early pages:

In order to assess the performance of DPP, an extensive study of experiments is :onducted using the 15-year trading data of Taiwan Stock Exchange Capitalization Weighted Stock Index. The result shows that DPP outperforms the existing predictive

which can be argued it contains a complete economic cycle, at least from the Western economic crisis perspective back to "bull markets". Taiwan had a deep as well around 2008 as seen in the picture below. Please, analysis, both Asian and Western stock markets and discuss the model outcome and performance, together with other models, as repeated requested above.

Some models are good for certain periods than others and it is a good idea to get a grip of the propose model "best time to be used" within an economic cycle, if it has any. In the discussion, please, include which model is good in which period within an economic cycle, etc. Still, the above 3% claim cannot necessarily be attributed that the market was particularly good to predict (as seen below in the figure); other reasons may be behind such results. Therefore, comparison with other models and data sources and time frame could clarify the issue.

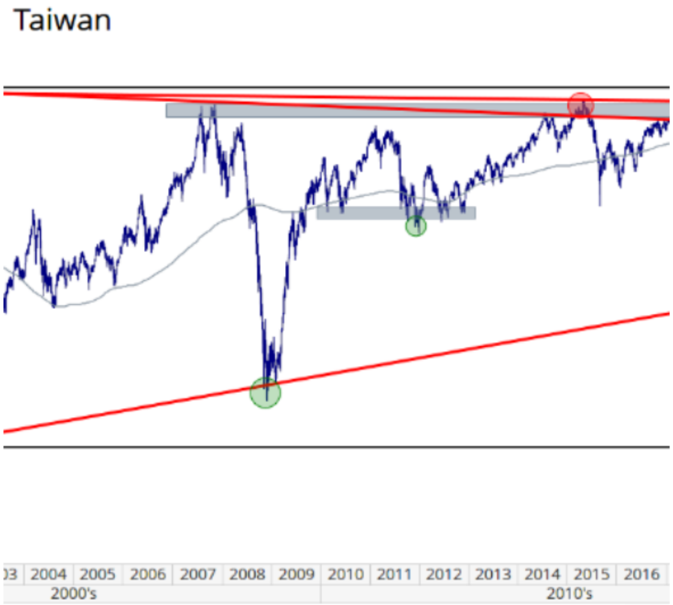

Thank you very much for the suggestions. Instead of using a stock market index in the Western stock market, we decided to use another stock market index in the Asia stock market: Nikkei 225 for the Tokyo Stock Exchange. NI225. The choice of this data set is based on the similarities between the economies and cultures of Taiwan and Japan as well as sharing the same color theme in the candlesticks (Red for up and Green for down) while the Western stock markets use different color themes in the candlesticks. Besides, regarding the daily price fluctuation limit, TAIEX imposes no daily price fluctuation limits which is consistent with the major markets in Europe and the United States (https://www.tpex.org.tw/web/stock/trading/information/range.php?l=en-us) whereas the daily fluctuation limit in Japan stock market is decided by the interval of stock price.

While it would be good to analyze the Asian and Western stock markets, the Taiwan stock market including TAIEX is highly associated with the Western stock markets especially the U.S.

stock markets (Aggarwal and Rivoli 1989) as the Taiwan stock market is often affected by the political as well as financial decisions made by the U.S. government. Our model sensitivity study also supports that the performance of DPP based models has some merits when some specific price patterns occur.

Therefore, we believe that selecting Nikkei 225 for the Tokyo Stock Exchange for the comparison with the results obtained based on the TAIEX index seems to be a fair decision as they have some similar patterns as well as some inconsistent patterns over time (See Fig 6 attached below).

Reference: Aggarwal R, Rivoli P. The relationship between the US and four Asian stock markets. ASEAN Economic Bulletin. 1989 Jul 1:110-7.

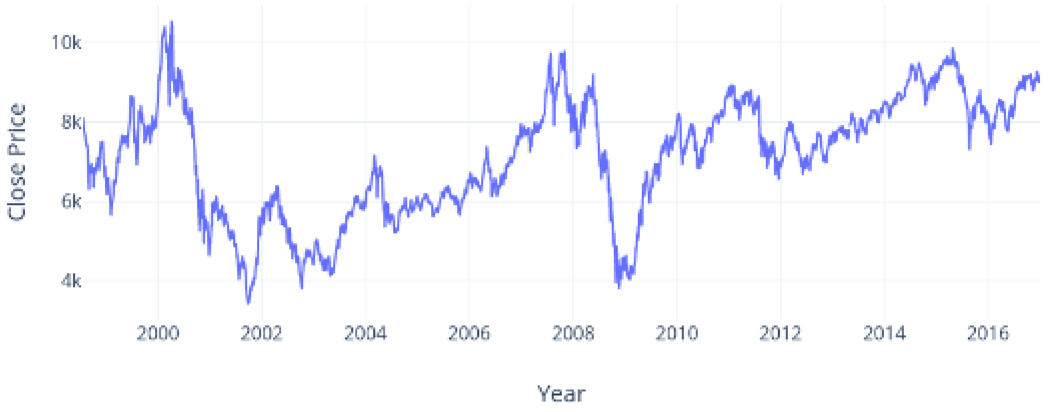

(a) TX dataset

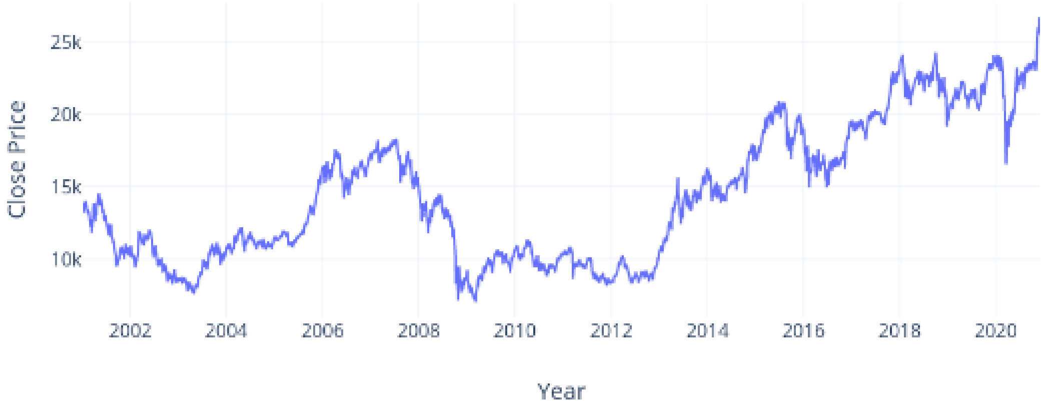

(b) NI225 dataset

Fig 6. Close price over years

12- Please, plot the outcomes of the methods to their targets, to visualize claims like "achieves higher accuracy".
Thanks

Since the outcomes of the methods are the predicted values of their target values and the possible values for them would be 0 or 1. In the figure below, we plot the results of the ground-truth in blue and those of prediction in red based on the DPP based model using the TX dataset in 2015. For each result, if it is upward on day t, there is a bar above x-axis at x=t. Otherwise, there is a bar below x-axis at x=t. By comparing multiple such figures it seems to be hard to visualize how the higher accuracy was achieved. We included Fig 10 and Fig 11 in the manuscript when studying the sensitivity of the accuracy values due to different selections of hyper-parameters, these two figures would be sufficient to show how we achieved a relative higher accuracy among all combinations considered.

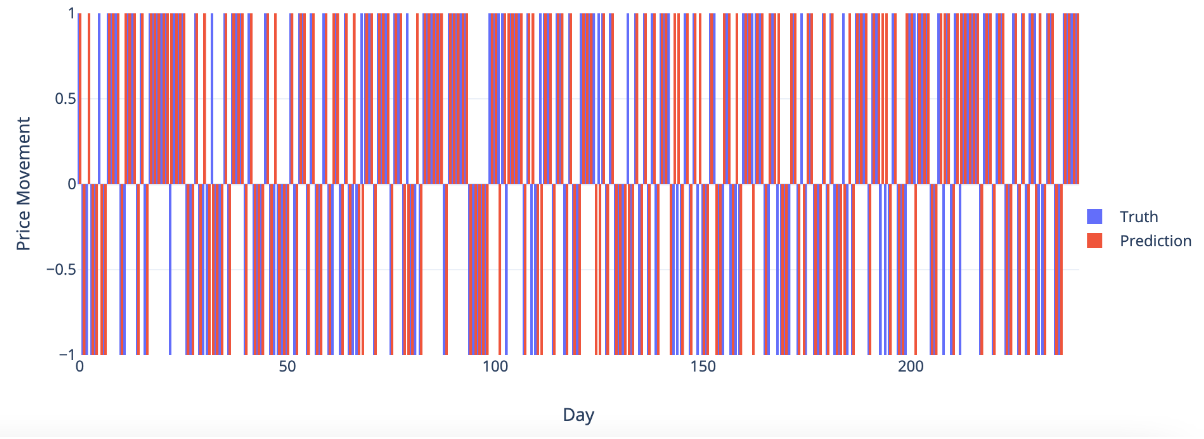

We thank you for your constructive feedback and comments. The paper was significantly revised and repositioned to emphasize the contribution of our work. We believe that this revised manuscript addresses the important points brought in this review.
